# Human Infection with *Burkholderia thailandensis*, China, 2013

**DOI:** 10.3201/eid2308.170048

**Published:** 2017-08

**Authors:** Kai Chang, Jie Luo, Huan Xu, Min Li, Fengling Zhang, Jin Li, Dayong Gu, Shaoli Deng, Ming Chen, Weiping Lu

**Affiliations:** Third Military Medical University, Chongqing, China (K. Chang, J. Luo, H. Xu, M. Li, F. Zhang, J. Li, S. Deng, M. Chen, W. Lu);; Shenzhen Academy of Inspection and Quarantine, Guangdong, China (D. Gu)

**Keywords:** Burkholderia thailandensis, comparative genomics, China, bacteria, virulence

## Abstract

*Burkholderia thailandensis* infection in humans is uncommon. We describe a case of *B. thailandensis* infection in a person in China, a location heretofore unknown for *B. thailandensis*. We identified the specific virulence factors of *B. thailandensis*, which may indicate a transition to a new virulent form.

*Burkholderia thailandensis* is closely related to *B. pseudomallei*, the causative agent of melioidosis (*1*). *B. thailandensis* shares most virulence factors and extensive genomic similarity with *B. pseudomallei* but can be distinguished by its ability to assimilate arabinose and different rRNA sequences (*2*,*3*). Little is known about *B. thailandensis* infection in humans. Two case reports described soft tissue infection and pneumonia with sepsis in Thailand and the United States (*4*,*5*). We describe a clinical investigation of human infection with *B. thailandensis* in Chongqing, China.

In October 2013, a 67-year-old man in Chongqing was hospitalized with a 13-day history of fever, productive cough with white sputum, and shortness of breath. Symptoms had not improved after antimicrobial drug treatment at a local clinic. The patient denied contact with any sick persons and any environmental exposure. Empirical treatment with meropenem was used to prompt resolution of the patient’s symptoms before culture results were received. During the 6-day treatment course, the patient was transferred to Chongqing Infectious Disease Hospital for treatment. Subsequently, his general condition worsened, and his family wished to have him close to home. He was discharged and died 2 days later.

Laboratory evaluations of blood samples performed at the time of the patient’s admission showed a leukocyte count of 20.72 × 10^9^ cells/L with a markedly elevated 91.5% neutrophils, aspartate aminotransferase level of 75.5 U/L (reference range 15.0–40.0 U/L), alanine aminotransferase level of 85.0 U/L (reference range 9.0–50.0 U/L), interleukin-6 level of 352.1 pg/mL (reference range 0–7 pg/mL), and procalcitonin level of 24.37 ng/mL (reference range 0–0.25 ng/mL). A computed tomography scan of the patient’s chest showed a thick-walled cavitary lesion at the posterior segment of the right upper lobe measuring 7.9 × 6.1 cm and multiple nodules in both lung fields (Technical Appendix Figure 1).

On day 6 of the patient’s hospitalization, we observed via microscopy that the positive blood culture contained many gram-negative rod-shaped bacteria (Technical Appendix Figure 2, panel A). The colonies were smooth and glossy, with silver pigmentation, on sheep blood agar Technical Appendix Figure 2, panel B). The VITEK 2 COMPACT system (bioMérieux, Marcy L’Étoile, France) identified the isolated strain as *B. pseudomallei* (97% probability; bionumber 0003451513500211). The API 20NE system (bioMérieux) also identified the isolated strain as *B. pseudomallei* (50.5% probability; index 1157577). However, the biochemical profiles of the API 20NE system, including arabinose assimilation, identified the isolated strain as *B. thailandensis*, based on the mode of artificial interpretation. We analyzed the 16S rDNA sequence of strain BPM with nucleotide BLAST (https://blast.ncbi.nlm.nih.gov/Blast.cgi) and found a 100% similarity with *B. thailandensis* (GenBank accession nos. CP000085.1 and CP000086.1). 

These results indicate that commercially available phenotypic assays are not ideal for the identification of *B. thailandensis*, which has not yet been incorporated into the databases of identification systems (*6*,*7*). Moreover, the arabinose assimilation proved to be an effective, simple, and accurate method for differentiating *B. thailandensis* from *B. pseudomallei*. When *B. pseudomallei* is presumptively identified, arabinose assimilation should be emphasized in clinical laboratories.

We compared the virulence of the isolated strain with *B. thailandensis* E264 (strain ATCC 700388) in BALB/c mice. *B. thailandensis* E264 is an environmental isolate from northeast Thailand. The clinically isolated *B. thailandensis* from this study was defined as strain BPM. Groups of 5 mice were inoculated with 10^7^ CFU of each isolate and observed for a period of 7 days after infection. Four fifths of the mice infected with strain BPM died within 1 week of challenge. *B. thailandensis* could be isolated from the bloodstream of mice at the time of death. In contrast, all mice with *B. thailandensis* E264 infection survived over a 1-week monitoring period (Figure, panel A). The histologic findings were notable for early dissemination to the liver and lung (Figure, panel B). We observed multiple large, necrotizing foci in the livers of mice infected with strain BPM and alveolar-based neutrophilic inflammation in the strain BPM infection group. In addition, the inflammatory infiltrate and lung hyperemia were raised in the BPM-infected mice. This finding is consistent with the clinical case in our study, which appeared as pneumonia and sepsis. Overall, these experiments confirm that strain BPM is a virulent pathogen.

**Figure:**
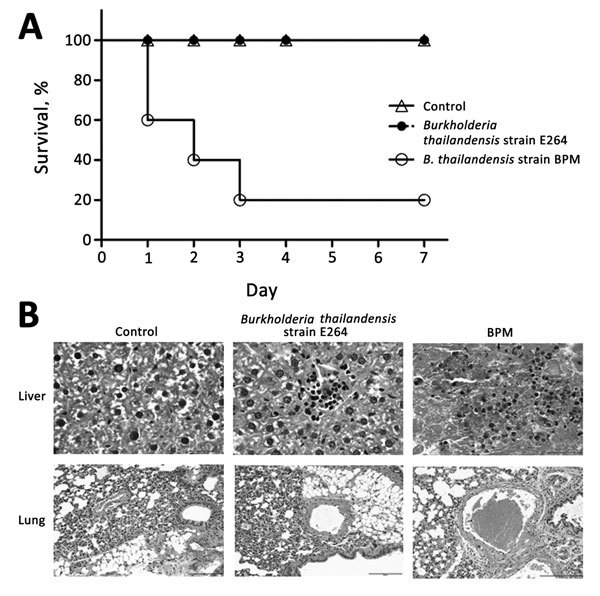
Virulence comparison of *Burkholderia thailandensis* isolated from a man in Chongqing, China, compared with *B. thailandensis* E264 (strain ATCC 700388). A) Survival pattern of 5 BALB/c mice intraperitoneally challenged with 10^7^ CFU and followed up for 7 days after challenge. B) Histopathologic characteristics of *B. thailandensis* intraperitoneal infection in the mice. Sections were stained with hematoxylin and eosin (original magnification ×40).

We performed comparative genomics to reveal the pathogenic mechanism of strain BPM. The BPM strain and *B. thailandensis* species share a large proportion of virulence factors. When compared with the reference genome sequences of *B. thailandensis* E264, *B. thailandensis* 2002721723, and *B. thailandensis* E444, the specific virulence factors of VirB/VirD4 type IV secretion system, HSI-I, and WcbR were indicated in strain BPM (Technical Appendix Table) (*8*–*10*). These specific virulence factors may represent a transition toward a new virulent form.

In conclusion, when considering *B. pseudomallei* infection, clinicians should also consider the possibility of *B. thailandensis* infection. *B. thailandensis* is not identified with use of commercially available phenotypic assays and may be mistaken for *B. pseudomallei*. In the future, deep analysis of the complete genome would be helpful in understanding the evolution of *B. thailandensis* and its adaptation to the environment.

Technical Appendix Virulence factors of *Burkholderia thailandensis* strain BPM and results of computed tomography scan of patient’s chest and of bacterial cultures.
