# Supplementary material for: Human Infection with Burkholderia thailandensis, China, 2013
Source: Emerg Infect Dis. 2017 Aug;23(8):1416–8. doi: 10.3201/eid2308.170048 (PMC5547772; doi:10.3201/eid2308.170048)
Supplement: Technical Appendix — Virulence factors of Burkholderia thailandensis strain BPM and results of computed tomography scan of patient’s chest and of bacterial cultures. [file 17-0048-Techapp-s1.pdf]

Article DOI: https://doi.org/10.3201/eid2308.170048

Human Infection with *Burkholderia thailandensis*, China

Technical Appendix

Technical Appendix Table. Specific virulence factors of *Burkholderia thailandensis* strain BPM

Gene_ID	Location	Subject_ID	Virulence factors	Description
BPMGL 000044	Scaffold1 53896–54636	VFG2240	VirB/VirD4 type IV secretion system	VirB11–VirB11 protein homolog
BPMGL 003055	Scaffold2 916394–919261	VFG2063	HSI-I	IcmF1-hypothetical protein
BPMGL 004458	Scaffold3 480105–482003	VFG2548	WcbR	Capsular polysaccharide biosynthesis fatty acid synthase

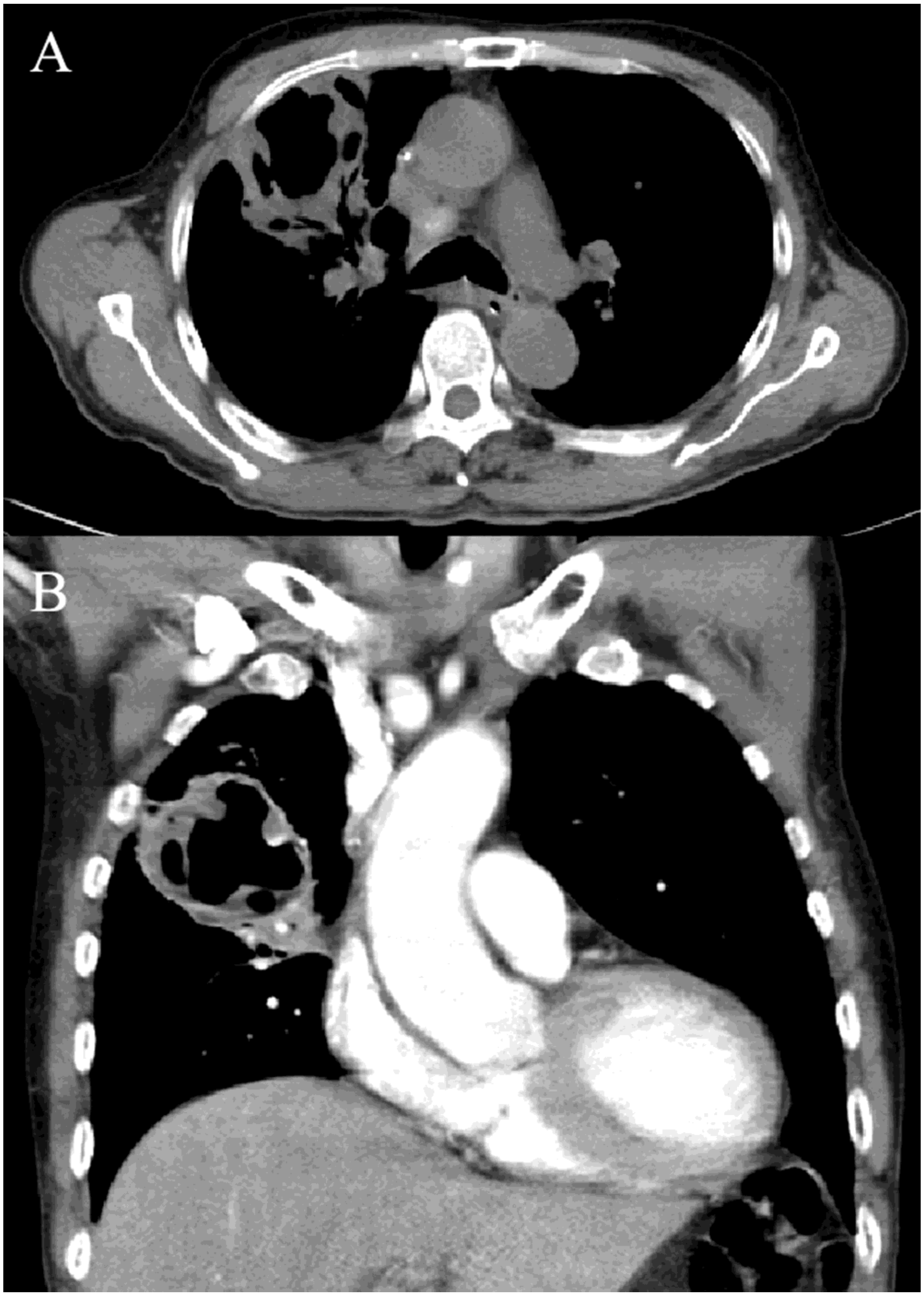

Technical Appendix Figure 1. Computed tomography scan of patient's chest showing a thick-walled cavitary lesion in the right chest. A) Cross section. B) Longitudinal section.

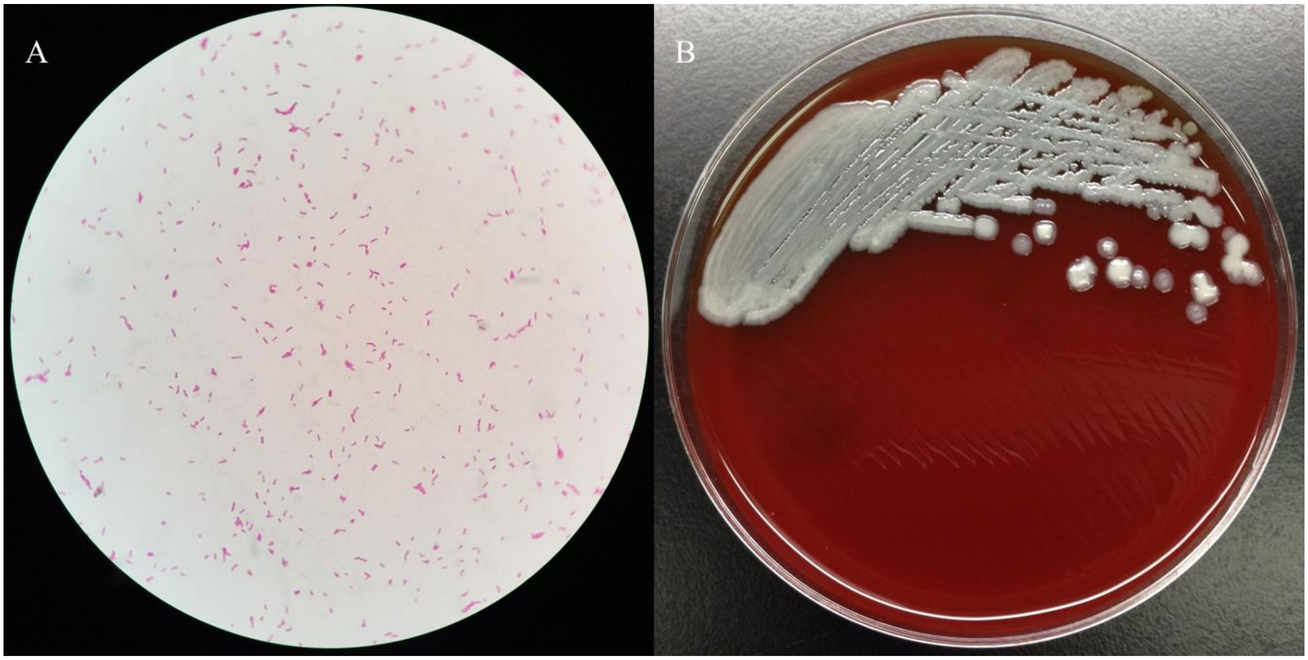

Technical Appendix Figure 2. A) Smooth and glossy colonies of *Burkholderia thailandensis* with a silver pigmentation on a blood agar plate. B) The gram-negative rod-shaped bacilli were observed by using Gram staining (original magnification ×1,000).
